# Is opium use associated with an increased risk of lung cancer? A case-control study

**DOI:** 10.1186/s12885-020-07296-0

**Published:** 2020-08-26

**Authors:** Ahmad Naghibzadeh-Tahami, Maryam Marzban, Vahid Yazdi-Feyzabadi, Shahryar Dabiri, Shokrollah Mohseni, Reza Abbasi Rayeni, Mitra Samareh Fekri, Mohammad Hasan Larizadeh, Behnaz Karimpour, Narges Khanjani

**Affiliations:** 1 Physiology Research Center, Kerman University of Medical Sciences, Kerman, Iran; 2 The Persian Gulf Tropical Medicine Research Center, The Persian Gulf Biomedical Sciences Research Institute, Bushehr University of Medical Sciences, Bushehr, Iran; 3 Social Determinants of Health Research Center, Institute for Futures Studies in Health, Kerman University of Medical Sciences, Kerman, Iran; 4 Pathology and Stem Cell Research Center, Department of Pathology, Afzalipour School of Medicine, Kerman University of Medical Sciences, Kerman, Iran; 5 Social Determinants in Health Promotion Research Center, Hormozgan Health Institute, Hormozgan University of Medical Sciences, Bandar Abbas, Iran; 6 Endocrinology and Metabolism Research Center, Institute of Basic and Clinical Physiology Sciences, Kerman University of Medical Sciences, Kerman, Iran; 7 Cardiovascular Research Center, Institute of Basic and Clinical Physiology Sciences, Kerman University of Medical Sciences, Kerman, Iran; 8 Neuroscience Research Center, Institute of Neuropharmacology, Kerman University of Medical Sciences, Kerman, Iran; 9 Social Determinants of Health Research Center, Institute for Future Studies in Health, Kerman University of Medical Sciences, Kerman, Iran; 10 Environmental Health Engineering Research Center, Kerman University of Medical Sciences, Kerman, Iran; 11 Monash Centre for Occupational & Environmental Health, School of Public Health and Preventive Medicine, Monash University, Melbourne, Australia; 12 Department of Epidemiology and Biostatistics, Faculty of Public Health, Kerman Medical University (KMU), Haft Bagh Alavi Highway, Kerman, 76169-11317 Iran

**Keywords:** Opioids, Risk factor, Lung neoplasm, Case-control

## Abstract

**Background:**

In recent years, lung cancer (LC) incidence has increased in Iran. The use of opium and its derivatives (O&D) has increased as well. This study aimed to investigate the association between the use of O&D and LC incidence.

**Methods:**

In this case-control study conducted in Kerman, Iran; 140 patients with lung cancer and 280 healthy controls matched by age, sex, and place of residence were included. Data, including O&D use, cigarette smoking, alcohol use, and diet, were collected using a structured questionnaire. The relation between the use of O&D and LC was evaluated using conditional logistic regression adjusted for tobacco smoking, education, daily intake of fruit, vegetables, red meat, and hydrogenated fats.

**Results:**

Opium ever-use was associated with an increased risk of LC (Adjusted Odds Ratio (AOR) =5.95, 95% CI: 1.87–18.92). Participants were divided into low and high use groups based on the median of opium use in the control group. A significant dose-response relation was observed between the amount of daily O&D use and LC; and the relation was stronger in high users (AOR _low users_ = 3.81% CI: 1.13–12.77 and OR _high users_ = 9.36, 95% CI: 2.05–42.72). Also, LC was higher among participants starting the use of O&D at younger ages (≤ 41 years old vs never users AOR = 8.64, 95% CI: 1.90–39.18) compared to those who started at an older age (> 41 years old vs never users, AOR = 4.71, 95% CI: 1.38–16.08). The association between opium, and lung cancer among non-smokers was OR: 6.50 (95% CI: 2.89 to 14.64).

**Conclusion:**

The results of this study show that opium use is probably a dose related risk factor for lung cancer.

## Background

Base on the data published by the United Nations Office of Drugs and Crime (UNODC), it was estimated that there were 29.2 million opioid users in the world in 2017, and the production of global opium will increase rapidly in the coming years [1]. Iran is located near Afghanistan, the most prominent opium-producing country [1], and has one of the highest statistics of opium use in the world. Studies have reported opium abuse from several parts of Iran; and it is considered as the most important type of drug abuse in this country [2].

Studies dated back to the 1970s have reported the carcinogenic effects of opium; and its relation with cancers such as esophagus [3, 4], stomach [5, 6] larynx [7], lung [8–10] bladder [11–18], colorectal [19, 20] and head and neck cancers [21]. Experimental studies have shown that this carcinogenic effect may be attributed to nitrogen-containing heterocyclic compounds derived from the pyrolysis of morphine [22].

Lung cancer (LC) has been among the deadliest world cancers [23]. The incidence of LC varies from 10 to 80 in 100,000 among men, and 5 to 38 in 100,000 among women [24]. The incidence of LC increased rapidly in the first half of the twentieth century; and in the late twentieth century, it became the first preventable cause of death in the world [25]. More than half of all LC cases occur in developing countries, including Iran [26]. In 2018, LC was the fourth most common cancer after breast, stomach, and colorectal cancer in Iran; and the age standardized rate (ASR) of lung cancer was 9.1 in 100,000 in both sexes, and 12.5, and 5.5 in 100,000 in Iranian males and females, respectively [24].

Meta-analyses have shown that smoking cigarettes is an important risk factor for LC with a pooled multiple-adjusted RR of 6.99 (95% CI: 5.09 to 9.59) in women and 7.33 (95% CI: 4.90 to 10.96) in men [27]. Other LC risk factors include environmental and occupational exposures, second-hand smoke, air pollution, heavy metals, and chemical exposures [28–30].

Kerman is located in the south-east of Iran, and has a high prevalence of O&D use, which is around 11 to 15% in the adult population [31, 32]. Although studies have been previously conducted about the relation between O&D use and LC in Iran, some of these studies have not included confounders such as smoking, alcohol, and diet [9]. This current case-control study was performed to investigate the relation between O&D use and LC in a population with a relatively high prevalence of opioid use and has included confounders that may affect this relation.

## Methods

### Samples and data source

In this matched case-control study, patients with LC whose pathologic information was available at the Cancer Registry of Kerman University of Medical Sciences, and were diagnosed between January 2014 and December 2017, were included.

A minimum sample size of 132 cases was calculated with an online source and according to Kelsey et al. [33], based on a previous study which reported an adjusted OR of 3.1 between smoking opium and lung cancer [9]. O&D use in the control population was assumed to be 18.6%, based on a sample from a cohort conducted in Kerman city on the adult population [34], the control to case ratio was 2, power = 0.8, and type one error = 0.05.

The patients’ information, including home address, telephone number, and cancer type, were extracted from their medical records. A telephone call was made to the patients and/or their families to ask for their consent to participate in the study. Then a specific date and time were set for an interview, and the individuals were then visited in person and interviewed.

The controls were selected from the cases’ neighborhood. The first two houses on the right side of the cases’ house were selected. Then, from these houses for each case, two neighbors that matched the inclusion criteria and were willing to cooperate, were enrolled as the controls. Both controls were matched for age (±5) and gender. If one of the neighbors was not present or did not want to cooperate, the next neighbor was approached. Data from the control group was gathered by face to face interviews, similar to the case group. The purpose of the study was explained to all participants, and written informed consent was taken before data collection.

### Instruments and data collection procedures

Data were collected using a questionnaire. The first part of the questionnaire consisted of demographic information including gender, age, education, and marital status, and the second part asked about the use of O&D, alcohol, cigarette smoking, and diet.

The researchers used a questionnaire for diet, which had been validated and used in previous studies [20, 35].

In order to quantify O&D use and cigarette smoking, the history of exposure in the past were asked. The cumulative lifetime use of opioids was calculated based on the amount and duration of use in different ages. Detailed questions about the age of starting opium use, daily amount and frequency of use (how many days a week, if weekly or more), routes of administration, opium types, and the age of quitting for those who had quit, were asked. Routes of usage included opium smoking, ingestion, or both. Opium types included *teriak*, *sukhteh*, and *shireh*. The validity and reliability of this questionnaire had been confirmed in a previous study. The reliability of opium use and duration of opium use was estimated to be 0.96 and 0.74, respectively. Furthermore, self-reported opium use had a sensitivity of 0.93 and a specificity of 0.90 [36].

The daily use of O&D was measured based on the local measurement unit *nokhod*, which is equal to 0.2 g. All data were converted to grams for more clarification. The type of opioids used was divided into four categories: raw opium (*teriak*), sap (*shireh*), burned opium (*sukhteh*), and heroin. However, no participant reported the use of heroin or burned opium.

A trained interviewer conducted all interviews, and in order to minimize under-reporting of O&D use, the interviewer explained the purpose of the study thoroughly and convinced participants that their information would be kept confidential. If the LC case was not alive, the closest family member would be interviewed.

### Data analysis

The cumulative consumption (median daily use × duration of consumption) was calculated for cigarette smoking, O&D, and alcohol use, and the participants were categorized into three groups: non-users, low users (≤ median use in the controls), and high users (>median use in the controls). The median use in the control group was considered as a criterion for classifying the population into high and low users. In all analyses, the non-user group was considered as the reference group.

A main concern for the association of opium and LC is reverse causality, because some patients had started using opioids after their symptoms had started. Therefore, opioid use after cancer diagnosis was not considered. Only O&D use from the past until 2 years before the diagnosis of lung cancer or enrollment into the study (for controls) was investigated.

In the case of food that was highly consumed, such as red meat, fruits and vegetables, solid oils and liquid and frying oils, we considered the frequency of weekly consumption, that is the number of times per week they consumed that food. Then we divided the data in two categories, according to the 50th percentile cut point. For example, for red meat, below the 50th percentile was less than 6 times a week and above the 50th percentile was more than 6 times a week. In case of food that was not frequently used, such as olive oil and other oils, they were divided into 2 groups, as sometimes consuming (Yes) or never consuming (No).

Conditional logistic regression with a 95% confidence level was used for data analysis. In order to control the effect of confounding variables, variables with a *P*-value of less than 0.1 in the univariate analysis were added to the final multivariate models. The confounding effect of specific dietary factors such as the use of meat, fruit, vegetables, hydrogenated fats, olive oil, as well as other exposures (cigarette and alcohol) and education were controlled. Multiplicative interactions between cigarette smoking (ever use) and opium use (ever use and cumulative dose) were evaluated using the likelihood ratio test.

Smoking and opium use were defined in three classes, which were never users, low dose users and high dose user. But, in some situations, where the numbers in the subgroups were too low, we used only two classes which were never uses and anytime (ever) users.

All statistical analyses were performed using STATA (version 14.0, Stata Corp, College Station, TX, USA). Statistical significance levels less than 0.05 were considered significant.

## Results

The initial number of people approached for this study was 174 cases and 348 controls. However, 34 patients did not consent to participate. The frequency of non-response was 19.5%. Eventually, 140 cases and 280 controls were included. Among the cases, the majority (68.57%) were male and married (96.43%).

Most of the cases (60.71%) were in the age range of 51–70 years at the time they entered the study. More than half of the cases were illiterate or had elementary education (62.14%). As controls were well matched for age and gender, no significant difference was observed in these variables between cases and controls (*P* > 0.05). But, education was significantly different between the two groups and was therefore adjusted in the statistical models. Demographic information about the cases and controls are presented in Table 1.

**Table 1:** Demographic variables in the case and control groups

Variable	Lung Cancers	Matched Controls	***P***-value*
N	140	280	
**Gender**			
Male	96 (68.57)	192 (68.57)	1.0
Female	44 (31.43)	88 (31.43)	
**Marital status**			
Married	135 (96.43)	273 (97.50)	0.53
Single	5 (3.57)	7 (2.50)	
**Age**			
≤ 50	35 (25.0)	78 (27.86)	0. 63
51–70	85 (60.71)	170 (60.71)	
> 70	20 (14.29)	32(11.43)	
**Education**			
Illiterate or Elementary Education	87 (62.14)	68 (24.29)	0.001
Middle or High school	42 (30.0)	138 (49.22)	
High School Diploma or above	11 (7.86)	74 (26.43)	

*P-values calculated according to McNemar’s test

The most common method of opioid use among the cases (93.0%) and controls (100%) was smoking. The median of opium use per day was 4.5 g, and the median of the duration of consumption was 20 years, in the control group. The median of cumulative opium use in the control group, was 87.5 g-years.

Table 2 shows the results of O&D use and LC. As shown in Table 2, 59.28% of the cases and 19.64% of the controls had a history of O&D use. O&D ever-use was significantly associated with an increased risk of LC (AOR =5.95, 95%CI: 1.87–18.92).

**Table 2:** The association between the use of opioid and its derivatives, and other variables (cigarette smoking and alcohol) and the incidence of lung cancer

Variable	Cases N (%)	Controls N (%)	Crude OR (95%CI)	Adjusted OR (95%CI)^a^
Opium use				
Never	57 (40. 71)	225 (80.36)	Reference	Reference
Ever	83 (59.28)	55 (19.64)	9.73 (5.21–18.15)	5.95 (1.87–18.92)
Amount of daily opium use				
Never used	57 (40.71)	225 (80.36)	Reference	Reference
≤ Median (4.5 g per day)^b^	36 (25.71)	29 (10.36)	6.83 (3.40–13.73)	3.81 (1.13–12.77)
> Median (4.5 g per day)	47 (33.57)	26 (9.29)	12.46 (5.76–26.92)	9.36 (2.05–42.72)
Duration of opium use				
Never used	57 (40.71)	225 (80.36)	Reference	Reference
≤ Median (20 years) ^b^	41 (29.29)	30 (10.71)	7.32 (3.70–14.49)	3.47 (1.13–10.62)
> Median (20 years)	42 (30.00)	25 (8.93)	12.98 (5.72–29.48)	5.50 (1.32–22.91)
Cumulative use of Opium^c^				
Never used	57 (40.71)	225 (80.36)	Reference	Reference
≤ Median (87.5 g-years)^b^	46 (32.86)	28 (10.00)	4.22 (1.44–12.30)	3.95 (1.29–12.12)
> Median (87.5 g-years)	37 (26.43)	27 (9.64)	8.60 (1.77–41.63)	4.79 (0. 88–26.08)
Age at start of opium use				
Never used	57 (40.71)	225 (80.36)	Reference	Reference
> Median (41 years)	22 (15.71)	27 (9.64)	5.69 (2.40–13.47)	4.71 (1.38–16.08)
≤ Median (41 years)^b^	61 (43.57)	28 (10.00)	11.90 (6.02–23.53)	8.64 (1.90–39.18)
Cigarette smoking				
Never	59 (42.14)	205 (73.21)	Reference	Reference
Ever	81 (57.86)	75 (26.79)	5.89 (3.30–10.50)	1.43 (0.50–4.10)
Daily cigarette smoking				
Never used	59 (42.14)	205 (73.21)	Reference	Reference
≤ Median (33 cigarettes per day) ^b^	23 (16.43)	40 (14.29)	3.19 (1.54–6.58)	1.10 (0.28–4.27)
> Median (33 cigarettes per day)	58 (41.43)	35 (12.50)	8.29 (4.35–15.79)	1.36 (0.43–4.34)
Duration of cigarette smoking				
Never used	59 (42.14)	205 (73.21)	Reference	Reference
≤ Median (30 years)^b^	40 (28.57)	42 (15.00)	4.74 (2.49–9.02)	1.09 (0.32–3.72)
> Median (30 years)	41 (29.29)	33 (11.79)	8.39 (3.90–18.06)	2.28 (0.59–8.77)
Cumulative use of cigarette smoking^c^				
Never used	59 (42.14)	205 (73.21)	Reference	Reference
≤ Median (49.5 pack-years)^b^	23 (16.43)	38 (13.57)	3.11 (1.52–6.40)	1.83 (0.49–6.73)
> Median (49.5 pack-years)	58 (41.43)	37 (13.21)	9.04 (4.60–17.70)	1.76 (0.57–5.42)
Age at start of cigarette smoking				
Never use	59 (42.14)	205 (73.21)	Reference	Reference
> Median (30 years)	62 (44.29)	61 (95.71)	5.45 (3.01–9.89)	4.71 (1.38–16.08)
≤ Median (30 years)^b^	19 (13.57)	14 (5.0)	8.48 (3.44–20.85)	8.64 (1.90–39.18)
Alcohol Use				
Never	135 (96.43)	277 (98.93)	Reference	Reference
Ever	5 (3.57)	3 (1.07)	3.33 (0.79–20.85)	2.34 (0.23–23.86)

^a^Specific dietary factors such as the use of meat, fruit and vegetables, hydrogenated fats, olive oil, as well as other main exposures (cigarette smoking and alcohol) and education were controlled. ^b^Median of use in the control group was considered as the cut off point. ^c^Cumulative use was obtained by multiplying the amount of use (per day) and the duration of use (per year)

A significant dose-response relation was observed between the amount of daily O&D use; and the relation was stronger in high users (AOR _low users_ = 3.81, 95%CI: 1.13–12.77 and AOR _high users_ = 9.36, 95% CI: 2.05–42.72).

Also, compared to the never used group, the use of O&D for more than 20 years significantly increased the risk of LC, and this association was significant even after adjusting (AOR: 5.50, 95% CI: 1.32–22.91).

Cumulative use of O&D increased the odds of LC, and showed a dose-response relation between O&D use and the incidence of LC.

The odds of LC in people who started to use O&D in younger ages (≤ 41 years old, versus never used, AOR: 8.64; 95% CI: 1.90–39.18) was higher than those who started at an older age (AOR: 4.71; 95% CI: 1.38–16.08).

About 57.86% of the cases were ever-smokers, while this rate was 26.79% for the controls.

High daily cigarette smoking (> 33 cigarettes per day) was associated with a significant increase in the incidence of LC (OR: 8.29; 95% CI: 4.35–15.79), but it did not remain significant after adjusting for confounders.

The interaction between cigarette smoking (ever use) and O&D use (ever use or cumulative dose) was not statistically significant (*P* = 0.38 and *P* = 0.14, respectively) in the logistic model.

In this study, the prevalence of alcohol use was low, and only 3.57% of the cases and 1.07% of the controls had a history of alcohol use. In the univariate analysis (OR 3.33; 95% CI: 0.79–20.85) and multivariate analysis (AOR: 2.34; 95% CI: 0.23–23.86), the odds ratios for alcohol use and LC were not significant.

There were 29 participants in this study who were opium users, but did not smoke. After splitting the data based on smokers and non-smokers, the crude association between opium, and lung cancer among smokers was OR: 3.27 (95% CI: 1.58 to 6.75), and among non-smokers was OR: 6.50 (95% CI: 2.89 to 14.64).

## Discussion

The results of this study showed that the majority of patients with lung cancer had a history of O&D use, and the use of O&D may be associated with an increased risk of LC. Also, in this study, there was a dose-response relation between O&D use variables and the risk of LC, and by increasing O&D use, the risk of this type of cancer increased as well. A study conducted previously in Tehran, Iran, showed that opium ever-use increased the odds of LC over seven times; and a higher frequency, longer duration and higher cumulative use (smoking or ingesting) of opium, showed a significantly increased risk of lung cancer. These researchers also found a significant (*p* < 0.001) positive trend in the association between lung cancer and duration of opium smoking or ingesting [9].

However, the relation between O&D use and cancer may be confounded by other risk factors such as age, gender, cigarette smoking, and alcohol use [37]. In Iran, opioid consumers are mainly older people who are more likely to be cigarette smokers as well [38]. However, after adjusting for confounding factors, including cigarette smoking, alcohol use, education, and diet, the relation between LC and opioids remained significant in our study.

Meanwhile, opioids may also be used after the establishment of cancer and in order to relieve pain. Therefore, in this study, in order to prevent reverse causality, the history of O&D use from the past until 2 years before the diagnosis of cancer was investigated, although many previous studies did not included a lag time for exposure data collection [4, 9].

Many mechanisms have been proposed about the carcinogenicity of O&D. Studies have shown that O&D and its alkaloids, including morphine, have mutagenic effects [22]. Empirical studies have shown that pyrolyzed opium has mutagenic effects on *salmonella* strains [39]. Also, pyrolysates and morphine alkaloids have led to sister chromatid exchange in human lymphocytes and morphological changes in cultured Syrian hamster embryo cells [40]. They have also caused carcinogenic changes after being injected under the skin, inside the trachea, or into the gastrointestinal system of rats [39]. It has also been shown that aromatic hydrocarbons released from burning opioids, indirectly lead to DNA damage and, as a result, may stimulate mutagenic mechanisms [41]. However, the carcinogenic mechanisms of opioids have not been thoroughly identified yet, and further studies are required.

It is worth mentioning that many chemicals are added to opioids during their processing, which may have carcinogenic effects, as well. One of these chemicals is lead, which is added by drug dealers to increase the weight of the product, and consequently increase their profit. In the studies conducted on opioids and addicted people in Iran, the amount of lead in the opioids and blood samples of addicts was much higher than usual and at levels that could cause severe health effects [42]. Studies have investigated the relation between occupational exposure to lead and the risk of lung cancer; and have shown that exposure to lead increases the risk of lung cancer by about three-fold, and the relation is dose-related [43].

In our study, we used the median of variables in the control group as the cut-point, because the median is robust to outliers. In many previous studies the median was used as the cut-point as well [4, 19, 21]. As shown in the results, the median of daily opium consumption among participants of the present study was 4.5 g per day, which is higher than previous studies. For instance, Shakeri et al. investigated the association between opium consumption, and pancreatic cancer and reported that the median of daily opium consumption was 0.4 g among participants in Tehran, Iran [37]; and in another study which was done among oesophageal squamous cell carcinoma (ESCC) cases and controls, the median of opium consumption per day was 1.5 *nokhod* or 0.3 g [4].

To the researchers’ best knowledge, there is a limited number of studies that have investigated the effect of opium on LC. The first study dates back to 1977 and was done in Singapore with 233 cases and 300 controls and showed an OR of 2.4 (95% CI: 1.4–4.0) [8]. After that, in 2012, a hospital-based case-control study with 242 cases and 484 matched controls conducted in Tehran, Iran, showed an adjusted OR of 3.1 (95% CI: 1.2–8.1). These authors also found that the concomitant use of opium and heavy cigarette smoking, dramatically increased the risk of lung cancer by an OR: 35.0 (95% CI:11.4–107.9) [9]. Another cohort study conducted in the northeast of Iran, showed that the opium use was associated with lung cancer, (OR 2.21, 95% CI: 1.44–3.39) in a dose dependent manner (p_trend_ < 0.05). These authors also showed that opium users have a significantly higher risk of developing cancers in different body organs, which one of them is the respiratory system [44]. Most of these studies confirm our results and show that opium and its derivatives can cause lung cancer.

Tobacco is the leading cause of LC in both men and women. In Iran, it is estimated that tobacco use accounts for more than 11,000 annual deaths in all ages, and smoking has had an upward prevalence over the recent decades [45]. Based on a survey that enrolled 5900 adults in Kerman, 8.3% of the study participants (15.5% of men and 0.8% of women) reported themselves as daily smokers [32]. As expected in this study, a significant association was found between smoking and LC. However, the significance of the association was lost after adjusting for confounders, including opium. This finding might mean that in this population opium is a risk factor, stronger than cigarette smoking for lung cancer; and because our sample size was not large enough, cigarette smoking did not become significant in the adjusted model. Also it was interesting to see the odds ratio for lung cancer and opium was stronger in the non-smoker (OR = 6.50) than the smoker (OR = 3.27) population.

One study conducted in Golestan, Iran about opium use and mortality showed a significant interaction between opium and smoking in relation to mortality [46]. However, in Mesjedi et al’s study about opium and lung cancer, due to zeros in the subgroups, evaluating the interaction of smoking cigarettes and smoking opium was not possible [9]. Besides in pancreatic cancer patients the interactions between cigarette smoking (ever use) and opium consumption (ever use or cumulative dose) were not statistically significant (*p* = 0.544 and 0.886, respectively) [47]. Our study did not find a significant interaction between opium use and cigarette smoking either.

In this study, we aimed to investigate the prevalence of alcohol consumption among participants. As the majority of Iranian people are Muslim, we did expect the prevalence of alcohol consumption to be much lower in these people than other parts of the world. A previous pooled analysis showed a weak relation between beer consumption and lung cancer, and the OR for ≥20 g/day users vs nondrinkers was 1.42; 95% CI: 1.06, 1.90 [48]. Alcohol consumption was included as a confounding variable in this study. However, we did not observe a significant relation, which may be explained by the low prevalence of alcohol users and non-existence of heavy alcohol drinkers in this study.

The dose response relation is one of the most important criteria for finding a causal relation between exposure, and outcome. In this study, participants with a cumulative use of ≤87.5 g-years opium had an adjusted OR of 3.95, but participants with > 87.5 g-years had an adjusted OR of 4.79. The dose-response relation was also observed in a previous study about O&D and LC [9]; and in a study on colorectal cancer patients, in which the OR was higher in high (OR = 7.7; 95% CI: 1.5–38.6) compared to low (OR = 3.4; 95% CI: 1.2–9.2) opium users [20]. Other studies have shown similar dose response relations between opium use and esophageal squamous cell carcinoma [4] and bladder cancer [18].

In this study, the minimum starting age of opium consumption in the control and case groups was 25 and 15 years, respectively. It is expected that people who start using opium at a younger age encounter the dangerous and mutagenic effects of this chemical more than others. In this study, we considered the never opium users as the reference group for comparison and found the adjusted odds ratio of 8.64 for people who started opium consumption sooner than others. Moreover, other studies have shown that the starting age of opium use might significantly affect the incidence of cancers such esophagus [4], and pancreatic cancers [37] as well.

Although in this study we used neighborhood controls, and we expected our participants to become matched for socio-economic status, but eventually we ended up with significant differences in educational status between cases and controls and we had to adjust for this variable. Similar to this, a study from India showed that as the level of education increased, the prevalence of opium use decreased [49].

One of the most critical challenges for any case-control study is to select the perfect control group. Most researchers agree, there is no ideal control group in a case-control study, and researchers need to think carefully about the representativeness of the control group, because a biased control group can lead to wrong results. In this study, we selected neighborhood controls because they were a better choice than hospital controls, as a variety of known and unknown diseases are caused by opium use. Also friend/family controls had the disadvantage of overmatching for opium use, as these habits prevail in specific social networks. Meanwhile, the interviewers who asked about exposure information were trained to explain for all participants that their information will remain confidential and reporting their opium use pattern correctly will not have any adverse consequences for them.

## Conclusion

This study showed that O&D use may be a dose related risk factor for lung cancer. This finding is consistent with many previous studies.

## Data Availability

The datasets generated during and/or analyzed during the present study can be inquired from the corresponding author on reasonable request.

## Abbreviations

LC: Lung cancer
O&D: Opium and its derivatives
AOR: Adjusted Odds Ratio
UNODC: United Nations Office of Drugs and Crime
CI: Confidence Interval
